# Effect of dragon fruit on glycemic control in prediabetes and type 2 diabetes: A systematic review and meta-analysis

**DOI:** 10.1371/journal.pone.0184577

**Published:** 2017-09-08

**Authors:** Nalinee Poolsup, Naeti Suksomboon, Naw Juna Paw

**Affiliations:** 1 Department of Pharmacy, Faculty of Pharmacy, Silpakorn University, Nakhon-Pathom, Thailand; 2 Department of Pharmacy, Faculty of Pharmacy, Mahidol University, Bangkok, Thailand; Florida International University Herbert Wertheim College of Medicine, UNITED STATES

## Abstract

**Objective:**

The purpose of this study was to systematically determine the effect of dragon fruit on glycemic control in prediabetes and type 2 diabetes.

**Methods:**

Electronic databases including MEDLINE, CENTRAL, CINAHL, Scopus, ScienceDirect^®^, Proquest, Web of Science^®^, LILACS, NAPRALERT, SciFinder, Clinicalkey, Herbmed, NCCIH and Google Scholar were searched from their earliest inception up to March 2017 for relevant randomized controlled trials (RCTs) which compared dragon fruit with placebo or no treatment in prediabetes or type 2 diabetes. Clinicaltrials.gov, clinicaltrialresults.org, and ISRCTN registry were also searched. Personal contact with experts and historical search of related articles was undertaken. Outcome of interest were fasting plasma glucose (FPG) and 2 hours post-prandial glucose (2HPP). Study selection, data extraction and study quality assessment were performed independently by two investigators. Disagreements were resolved by a third reviewer. Treatment effect was estimated with mean difference (MD). Effect estimates were pooled using inverse-variance weighted method. Heterogeneity was assessed with the Q statistic and quantified with the I^2^ statistic. DerSimonian and Laird random-effects model was used when the Q-statistic was significant at the level of 0.1, otherwise a fixed-effects model was used.

**Results:**

Among 401 studies identified from literature search, 4 RCTs involving 36 prediabetes subjects and 109 type 2 diabetes patients were included in the analysis. In prediabetes, FPG reduction was significant with MD of -15.1 mg/dL (95% CI: -23.8 to -6.5 mg/dL, P-value = 0.0006). Meta-analysis in type 2 diabetes showed no effect of dragon fruit on FPG (MD -26.5 mg/dL, 95% CI: -72.6 mg/dL to 19.6 mg/dL) and in 2HPP (MD -30.5 mg/dL, 95% CI: -80.9 mg/dL to 19.9 mg/dL).

**Conclusion:**

The available evidence in prediabetes is interesting. This will shed some light on diabetes prevention. The effect in T2DM was not significant. However, a trend towards greater blood glucose reduction with higher dose was observed.

## Introduction

In 2016, World Health Organization (WHO) reported that the incidence of diabetes all around the world is in an increasing trend since 1980, rising from 4.7% to a double 8.5% in 2014 in adult population. The majority of patients with diabetes are affected by type 2 diabetes [1]. Prediabetes is considered for individuals who have high risk for future development of diabetes. According to the American Diabetes Association, prediabetes is characterized by impaired fasting plasma glucose (FPG 100–125 mg/dl) or impaired glucose tolerance (IGT) (2 hours oral glucose tolerance test: OGTT 140–199 mg/dl) or HbA1C 5.7–6.4% [2]. General recommendations for controlling blood glucose level are dietary control, physical exercise and maintaining body weight. However, most people find it difficult to make such dietary and lifestyle changes. Therefore, pharmacologic intervention is often required.

Blood glucose level can be controlled by chemical drugs and medicinal plants which have an influence on blood glucose concentration effectively [3, 4]. Dragon fruit is one of the medicinal plants which has been reported to have a potential as diabetes mellitus treatment [5]. It is a climbing vine cactus species and commercially cultivated and marketed as *Hylocereus polyrhizus* (red skin with red flesh, red flesh dragon fruit, red pitaya), *Hylocereus costaricensis* (red skin with red purple flesh, red dragon fruit, red pitaya), *Hylocereus undatus* (red skin with white flesh, white flesh dragon fruit) and *Selenicereus megalanthus* (yellow skin with white flesh, yellow dragon fruit, yellow pitaya) [6, 7].

There is evidence from animal studies that dragon fruit has anti-diabetic effect by regenerating pancreatic-β cells and attenuating fibroblast growth factor-21 (FGF-21) resistance [8, 9]. Several non-randomized and randomized controlled trials have been conducted in order to assess the effectiveness of dragon fruit in patients with prediabetes [10, 11] and type 2 diabetes [12, 13, 14]. However, the results of these studies remain inconsistent and appropriate reviews which summarize the role of dragon fruit in diabetes management are required. This review was aimed to systematically evaluate the effect of dragon fruit on glycemic control in prediabetes and type 2 diabetes.

## Materials and methods

This systematic review followed the Preferred Reporting Items for Systematic Reviews and Meta-Analyses (PRISMA) guidelines. The protocol was not pre-registered.

### Search strategy

The following electronic databases were searched from their earliest records to the end of March 2017: MEDLINE (Pubmed), Cochrane Central Register of Controlled Trials (CENTRAL), Cumulative Index to Nursing and Allied Health Literature (CINAHL), Scopus, ScienceDirect^®^, Proquest, Web of Science^®^, Latin American and Caribbean Health Sciences Literature (LILACS), Natural Products Alert (NAPRALERT), SciFinder, Clinicalkey, Herbmed, National Center for Complementary Integrative Health (NCCIH), Google Scholar, https://clinicaltrials.gov/, http://www.clinicaltrialresults.org/ and International Standard Randomised Controlled Trials Number (ISRCTN) registry. The following medical subject headings (MeSH) terms including “betacyanin”, “diabetes mellitus”, “hyperglycemia”, “insulin resistance” were used. Title/abstract/keywords search was performed including [“*Hylocereus polyrhizus*” or “*Hylocereus undatus*” or “*Hylocereus costaricensis*” or “*Selenicereus megalanthus*” or “dragon fruit” or “dragon fruit juice” or “pitaya” or “pitahaya” or “betacyanin”] AND [“diabetes” or “prediabetes” or “insulin resistance” or “hypoglycemic effect” or “antidiabetic effect” or “blood glucose” or “impaired glucose tolerance”]. There was no language restriction. Hand search of related articles was undertaken. Personal contact with experts in the area was also carried out. Conference proceedings of the American Diabetes Association (ADA) and the European Association for the Study of Diabetes (EASD) were also scanned. Study authors and librarians were emailed for full text retrieval. Non-English language articles were translated into English.

### Selection of trials

Identified records were evaluated and selected independently by two reviewers, with a final review for eligibility was made by a third reviewer. Studies were selected if they were (i) randomized controlled clinical trials which compared the effect of dragon fruit with placebo or no treatment on glycemic control in patients with prediabetes or type 2 diabetes and (ii) reporting outcome measures in terms of fasting plasma glucose (FPG) or 2-hour post-prandial glucose (2-HPP) or glycated haemoglobin (A1C).

### Data extraction

Data from eligible studies were extracted and recorded independently by two reviewers. Disagreements were resolved by a third reviewer. The following data were extracted from each study: main author, year of publication, study design, study population, number of patients, duration of study, dragon fruit product and dose, baseline and endpoint values of outcome measure and details of treatment and control.

### Assessment of risk of bias

Two reviewers independently assessed the risk of bias as recommended by the Cochrane Handbook for Systematic Reviews of Interventions [15]. Disagreements were resolved by a third reviewer. The following methodological domains were considered: random sequence generation, allocation concealment, blinding of participants and personnel, blinding of outcome assessment, incomplete outcome data, selective reporting and other bias. The bias in each domain was judged as low risk, high risk and unclear risk of bias.

### Statistical analysis

Meta-analysis was conducted separately for prediabetes and type 2 diabetes. In prediabetes, treatment effect was estimated with mean difference in final value between the treatment and control groups. In type 2 diabetes, efficacy was assessed based on the change from baseline to final assessment. When the variances of these changes were not provided, they were computed using the following equation [15]:

$$SD\left(C\right)=\surd SD{\left(B\right)}^{2}+SD{\left(F\right)}^{2}–\left(2\times R\times SD\left(B\right)\times SD\left(F\right)\right)$$

where SD(C) is the standard deviation of change, SD(B) and SD(F) are standard deviations of baseline and final values, respectively. The correlation coefficient (R) of 0.5 was used [15]. Treatment effect was estimated with mean difference between the dragon fruit group and the control group. Both FPG and 2-HPP were reported in terms of mg/dL, A1C was reported as a percentage. The pooled mean difference and estimated 95% confidence interval were calculated using the inverse variance-weighted method. DerSimonian and Laird random- effects model was used when the Q-statistic for heterogeneity was significant at the level of 0.1, otherwise a fixed-effects model was used. The degree of heterogeneity was quantified using the I-squared statistic, which is the percentage of total variation across the studies due to heterogeneity. Sensitivity analysis was conducted using different statistical approaches, i.e. fixed-effects and random-effects methods. The Review Manager Software (RevMan 5.3.5) was used for data analysis. The significant level was set at P-value < 0.05. We planned to evaluate publication bias using funnel plot and Eggers’ test [16]

## Results

### Description of studies

**Fig 1:**
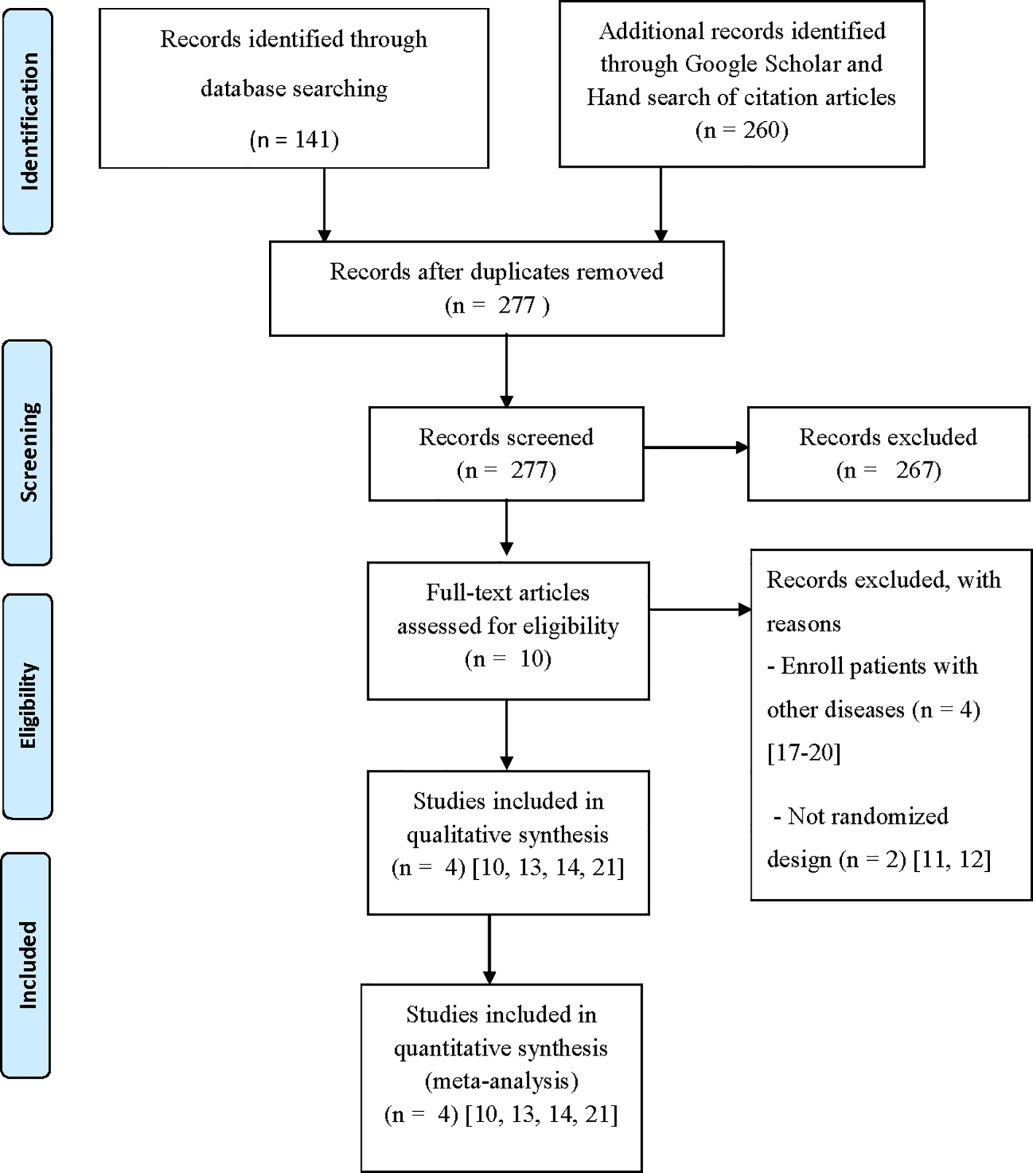
Study selection procedure.

Fig 1 shows the steps and number of trials identified and selected for inclusion. A total of 401 studies were identified from literature search. 124 duplicated records were removed. After screening titles and abstracts, 267 records of animal, phytochemical, food industry, agricultural, in vitro and review studies were excluded. Full texts of ten potential studies were assessed for eligibility. Four studies [17–20] were excluded because they enrolled patients with other diseases. Two studies [11, 12] were excluded for non-randomized design. Finally, four studies [10, 13, 14, 21] met the inclusion criteria and included in this systematic review and meta-analysis. All studies compared the effect of dragon fruit with no treatment. The preparations of dragon fruit used were red dragon fruit juice, fresh red dragon fruit and sprayed dried red dragon fruit powder. One study [10] was conducted in prediabetes and three studies [13, 14, 21] in type 2 diabetes. Three studies [10, 13, 21] reported FPG and one study [14] reported 2HPP as outcome measure. No study reported A1C outcome. Two trials [10, 21] were in the English language and two trials [13, 14] in the Indonesian language. Non-English studies were translated into English. The characteristics of the included studies and FPG and 2HPP outcome reported are summarized in Tables 1–3.

**Table 1:** Characteristics of the included studies in the systematic review.

Study (year)	Design	Species	Outcome measurement	Duration of treatment	Sample size	Study arms
Prediabetes						
Akhiruddin (2013)	Single-binded cross-over	*Hylocereus polyrhizus*	FPG	4 weeks	36	Mainly divided into PT and PC groups - PT group (n = 18) was subdivided into PT3, PT4, PT5 (n = 6/group) - PC group (n = 18) was subdivided into PC3, PC4, PC5 (n = 6/group)
Type 2 Diabetes						
Hadi (2012)	Non-blinded, Parallel	*Hylocereus polyrhizus*	FPG	4 weeks	28 (7:7:7:7)	Group 1: 400 g red dragon fruit/day Group 2: 600 g red dragon fruit/day Group 3: No treatment Group 4: Healthy subjects, no treatment
Hapsari (2015)	Non-blinded, Parallel	*Hylocereus costaricensis*	FPG	15 days	30 (15:15)	Group 1: 180 g red dragon fruit/day Group 2: No treatment
Wiardani et al. (2014)	Non-blinded, Parallel	*Hylocereus polyrhizus*	2HPP	10 days	51 (17:17:17)	Group 1: 100 g red dragon fruit juice/day Group 2: 200 g red dragon fruit juice/day Group 3: No treatment

Abbreviation: BMI = Body mass index, DBP = Diastolic blood pressure, FPG = Fasting plasma glucose, PT = Prediabetic treatment, PC = Prediabetic control, PT3 = 3 sachets or 60 g of red pitaya powder (RPP) drink/day, PT4 = 4 sachets or 80 g of RPP drink/day, PT5 = 5 sachets or 100 g of RPP drink/day, SBP = Systolic blood pressure, 2HPP = 2 hours post-prandial glucose

**Table 2:** Characteristics of the included studies in the systematic review.

Study (year)	Inclusion Criteria	Antihyperglycemic agents	Diet control and physical exercise	Compliance	Food recall
Prediabetes					
Akhiruddin (2013)	• FPG (5.6–6.9 mmol/L) • Age 25–55 years •BMI < 30 kg/m^2^ • SBP 100–140 mmHg, DBP 40–90 mmHg • Healthy, non-smoking, not taking drug, vitamin or supplement • No diabetes, coronary heart disease, cancer or other major illness	None	• Discontinue health supplement, vitamin C, fruits and juice • Routine physical exercise	Monitored but not reported	24 hours dietary intake for 3 days (2 weekdays and 1 weekend day)
Type 2 diabetes					
Hadi (2012)	• Type 2 diabetes (FPG ≥ 6.1 mmol/L) • Age 20–55 years • Non-pregnant, Non-drinkers	Taking oral antidiabetic medication	• No diet control and physical exercise	Not monitored	24 hours dietary intake for 3 days (2 weekdays and 1 weekend day)

**Table 3:** Characteristics of the included studies in the systematic review.

Study (year)	Inclusion Criteria	Antihyperglycemic agents	Diet control and physical exercise	Compliance	Food recall
Type 2 diabetes					
Hapsari (2015)	• Type 2 diabetes patients who were actively participating aerobic dance for elderly in polyclinic of program of chronic disease management • Age 47–78 years	Taking the pharmacologic therapy for diabetes mellitus	• Receiving diet program • Regular exercise every 2 weeks • Education once monthly	Monitored but not reported	3 times within study duration on day 1, 7, 15
Wiardani (2014)	• Type 2 diabetes • Age > 30 years • Not use insulin injection • Not use herbal supplement	Not reported	• Every juice intake was controlled • Receiving diet program	Monitored but not reported	24 hours food recall

### Risk of bias in included studies

**Fig 2:**
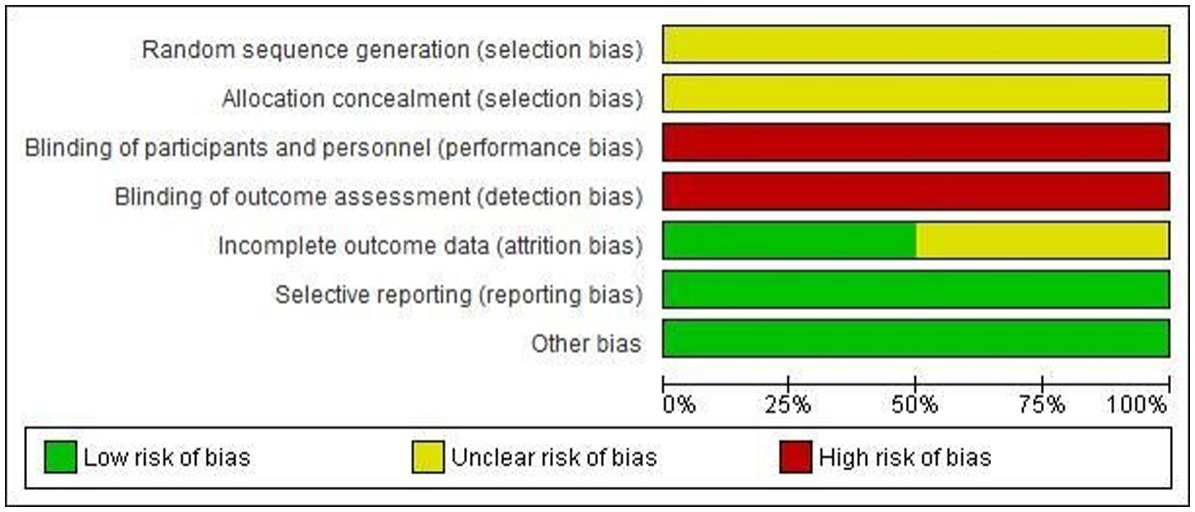
Risk of bias graph: Review authors' judgments about each risk of bias item presented as percentages across all included studies.

**Fig 3:**
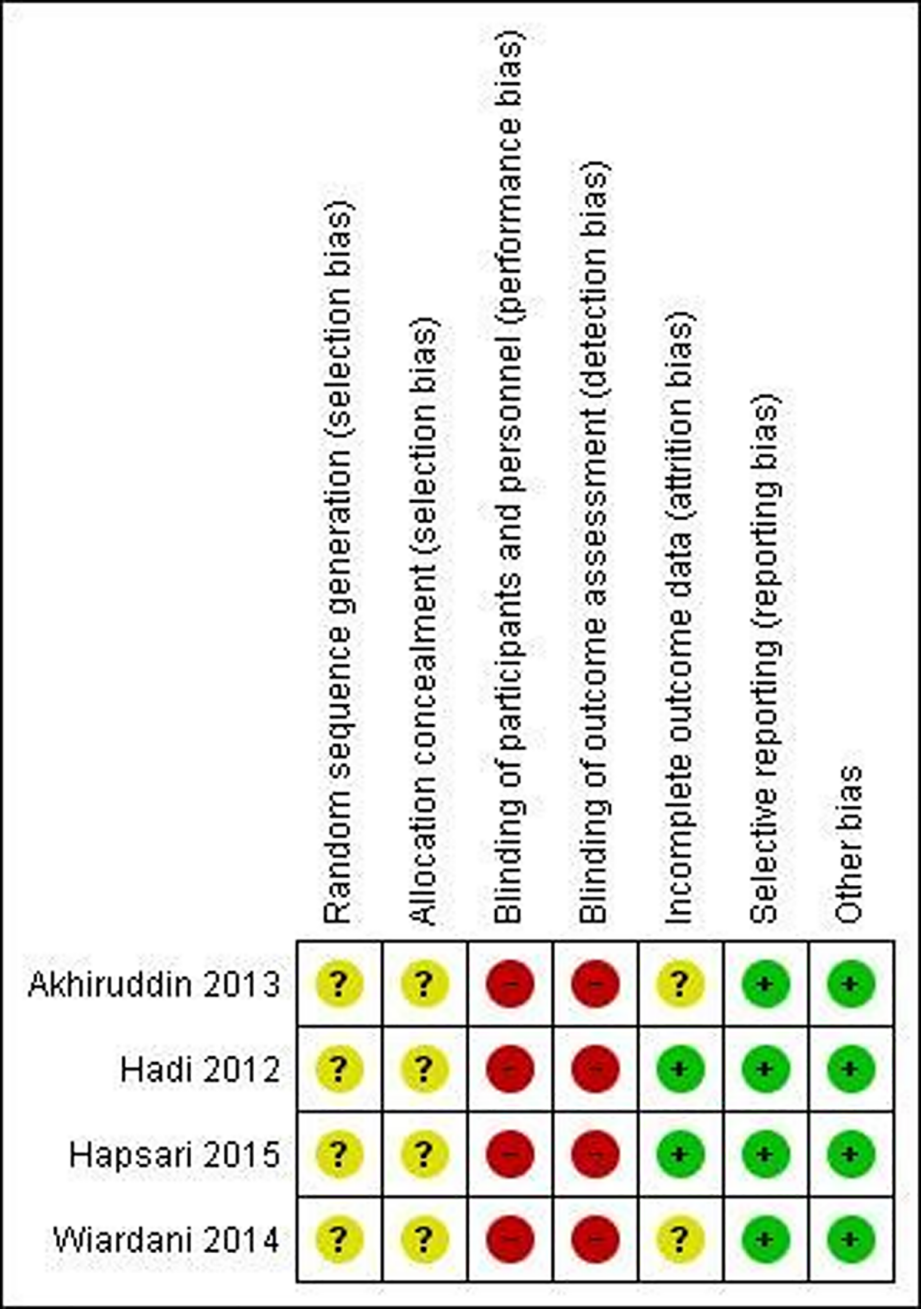
Risk of bias summary: Review authors' judgements about each risk of bias item for each included study.

The assessment of risk of bias is presented in the “Risk of bias graph” (Fig 2) and “Risk of bias summary” (Fig 3). All trials were described as randomized design but it was not clear how randomization was achieved as none of the trials described methods of random sequence generation and allocation concealment. Therefore, they were judged to have unclear risk of bias in random sequence generation and allocation concealment. Blinding of participants, personnel and outcome assessors were not undertaken in three studies [13, 14, 21] and only one trial [10] was described as single-blinded.

There was no attrition among two trials [13, 21], thus considered to be low risk of attrition bias. Among the remaining two studies [10, 14], it remained unclear. All trials reported outcomes pre-specified in the method section, being at low risk of reporting bias. Again, there was no other apparent risk of bias in all included studies.

### Effect of dragon fruit on glycemic control

#### Prediabetes

**Fig 4:**
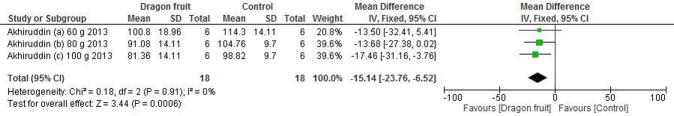
Forest plot of the effect of dragon fruit on FPG in prediabetes.

In the study by Akhiruddin, 2013, three treatment arms were compared with three different control groups. In such case, each comparison was treated as a separate study. The pooled result showed a significant reduction in FPG in favour of dragon fruit, with MD of -15.1 mg/dL (95% CI: -23.8 to -6.5 mg/dL, P-value = 0.0006) (Heterogeneity: Chi^2^ = 0.2, df = 2; I^2^ = 0%, 95% CI: 0–62%) (Fig 4). The effect remained unchanged when sensitivity analysis was conducted using the random effects model (MD -15.1 mg/dL, 95% CI: -23.8 to -6.5 mg/dL, P-value = 0.0006). The figure from fixed and random effects for prediabetes are exactly the same. Perhaps because the data are from only one study.

#### Type 2 diabetes

**Fig 5:**
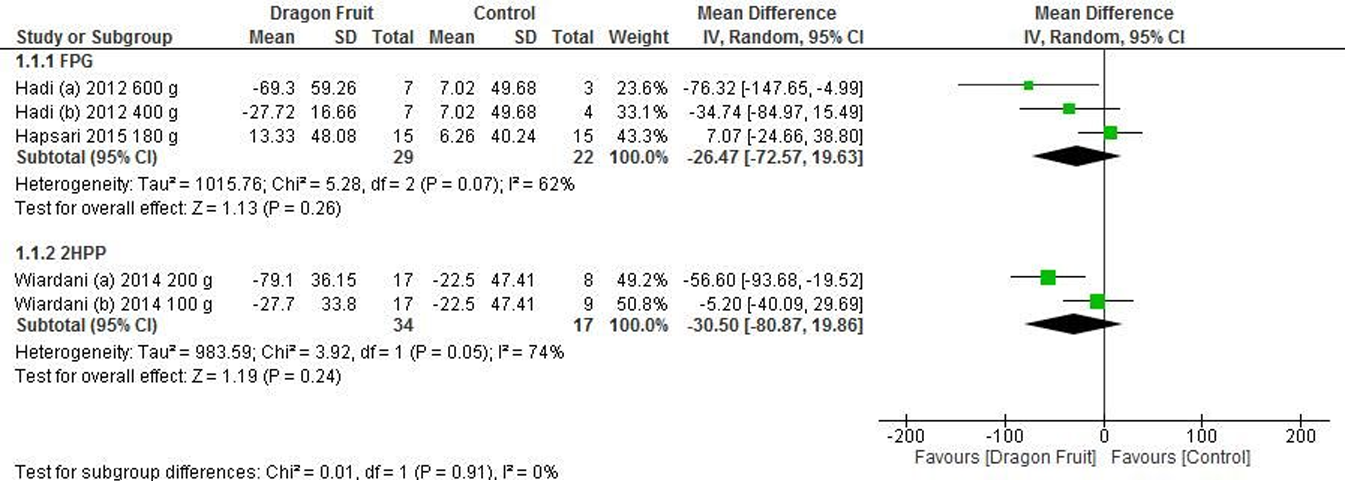
Forest plot of the effect of dragon fruit on FPG and 2HPP in type 2 diabetes.

Three studies [13, 14, 21] contributed data for meta-analysis. Analysis was conducted separately for FPG and 2HPP. Two treatment arms were compared with a single control group in two studies [14, 21]. In such case, each comparison was treated as a separate study and the number of patients in the control group was divided into half in order to avoid double counting. The pooled result showed that FPG reduction was non-significant, with MD of -26.5 mg/dL [95% CI: -72.6 to 19.6 mg/dL, P-value = 0.26] (Heterogeneity: Chi^2^ = 5.3, df = 2; I^2^ = 62%, 95% CI: 0–89%). Again, the effect on 2HPP was not significant, with MD of -30.5 mg/dL (95% CI: -80.9 to 19.9 mg/dL, P-value = 0.24) (Heterogeneity: Chi^2^ = 3.9, df = 1; I^2^ = 74%, 95% CI: 0–94%). (Fig 5) Assessment for publication bias was not possible due to the reduced number of studies included in the meta-analysis.

Sensitivity analyses using fixed effects model revealed a favourable effect on 2HPP (MD -29.3 mg/dL, 95% CI: -54.8 to -3.9 mg/dL, P-value = 0.02), but the effect on FPG remained unaffected (MD -13.7 mg/dL, 95% CI: -38.8 to 11.4 mg/dL).

## Discussion

Dragon fruit is a rich source of natural antioxidants including betacyanin, flavonoid, phenolic acid, ascorbic acid and fiber [22–24]. With high antioxidant and free radical scavenging activity, it has preventive effect on histopathological picture of pancreatic β cells in alloxan induced diabetes rats by reducing reactive oxidative species [8, 25, 26]. White pitaya juice and purified peel betacyanins (PPBNs) improved insulin resistance through decreasing fibroblast growth factor-21 (FGF-21) expression and increasing level of FGF-21 related genes (Klb, FGFR2, Egr1 and cFos) in the liver [9, 27].

In the meta-analysis in prediabetes, the overall estimated treatment effect of dragon fruit on FPG was significant. However, in type 2 diabetes, the overall estimated effects on FPG and 2HPP were not significant; however, there was a trend towards greater glucose reduction achieved with higher dose since estimated treatment effect became greater with the higher dose. Of these, it might be indicated the potential benefit of dragon fruit. However, clinical trials in type 2 diabetes which compared different dosages of dragon fruit by multiple comparison analysis are still required to confirm this. Apart from this, there was a significant heterogeneity (P-value 0.07) between the two studies [13, 21] measured FPG. Although one study [13] offered diet program, education, physical exercise and monitored the compliance of consumption, the other study [21] did not. Even though the population of these two studies had apparently comparable baseline blood glucose level, body mass index, gender ratio and food intake, they were different in age (mean age 45.1±11.4 vs. 63.9±8.5 years). Differences in dragon fruit species tested, dosage and age of patients might contribute to some extent to the observed the heterogeneity.

All studies continued medication and usual care for diabetes during the study period and thus it is unclear whether the antihyperglycemic effect derived from dragon fruit merely. One study [21] carried out the wash out period and determined blood glucose level after removal of dragon fruit. It reported that blood glucose level increased, ranging from 5.3 to 10.2% after treatment was completely stopped. This study also compared the treatment group with healthy subjects group to reveal the efficacy of dragon fruit by comparing with active healthy control group. However, the results showed that the final mean blood glucose level of treatment group was not comparable with that of healthy subjects group. No adverse effect was reported in these studies and none of them measured long term safety.

This review attempted to evaluate the effect of both red and white dragon fruit on glycemic control in prediabetes and diabetes. However, only clinical studies in two species (*Hylocereus polyrhizus* and *Hylocereus costaricensis*) of red flesh dragon fruit were identified and included. This may be explained by the fact that red flesh dragon fruit has greater content of antioxidant compared with white flesh dragon fruit. The results of antioxidant activity tests, total phenolic content and total betacyanin content was highest in red peel, followed by white peel, red flesh and white flesh [28, 29]. Since glucose lowering effect of dragon fruit is possibly derived from betacyanin and antioxidant activity of dragon fruit, the effectiveness of red and white flesh dragon fruit may be different. Therefore, the clinical studies are required to confirm this.

Overall, the included studies had moderate risk of bias. Most of them lacked the clarity of methodological information for random sequence generation, allocation concealment, double-blinding and withdrawal or dropouts which are essential for assessment of risk of bias. There is evidence that unclearly concealed or non-blinded trials yield larger estimate of treatment effect (odd ratio exaggerated by 30% and 17% respectively) compared with trials in which authors reported adequately on concealment and double-blinding [30].

To our knowledge, this is the first meta-analysis and we included not only the studies published in medical journals but also unpublished academic research. Both English and non-English language studies were considered as well. However, limitations of this meta-analysis should be highlighted. Firstly, although extensive search was undertaken for published materials, the possibility of unpublished studies with negative findings cannot be excluded. Funnel plot asymmetry was not assessed because of the small number of studies included in the meta-analysis. Secondly, only four RCTs were involved and there were relatively few subjects in each study, thus may lack statistical power. Finally, the pooled estimated treatment effect in type 2 diabetes should be interpreted with caution because there was a high clinical heterogeneity between studies and overall poor quality of the included trials. As the available evidence is limited, further high quality, well-designed larger randomized controlled trials are required and HbA1C should be commonly measured.

## Conclusion

The available evidence of the effect of dragon fruit in prediabetes will be of interest in diabetes prevention. Nonetheless, further studies are warranted. The effect in T2DM was not significant but there was a trend towards greater blood glucose reduction with higher dose. Due to restricted available data and poor quality of clinical evidence, larger, adequate-power, well-controlled clinical trials are required to further evaluate the clinical benefit of dragon fruit in these patients.

## Supporting information

S1 Checklist (DOC)
